# Altered intrinsic brain activity and connectivity in unaffected parents of individuals with autism spectrum disorder: a resting-state fMRI study

**DOI:** 10.3389/fnhum.2022.997150

**Published:** 2022-09-30

**Authors:** Xiang-Wen Zhu, Li-Li Zhang, Zong-Ming Zhu, Luo-Yu Wang, Zhong-Xiang Ding, Xiang-Ming Fang

**Affiliations:** ^1^Department of Radiology, Affiliated Hangzhou First People’s Hospital, Zhejiang University School of Medicine, Hangzhou, China; ^2^Department of Child Health Care, Wuxi Children’s Hospital, Wuxi, China; ^3^Department of Radiology, Affiliated Wuxi People’s Hospital, Nanjing Medical University, Wuxi, China

**Keywords:** autism, unaffected parents, resting-state, functional magnetic resonance imaging, brain activity, functional connectivity

## Abstract

**Objectives:** Autism spectrum disorder (ASD) is a juvenile onset neurodevelopmental disorder with social impairment and stereotyped behavior as the main symptoms. Unaffected relatives may also exhibit similar ASD features due to genetic factors. Although previous studies have demonstrated atypical brain morphological features as well as task-state brain function abnormalities in unaffected parents with ASD children, it remains unclear the pattern of brain function in the resting state.

**Methods:** A total of 42 unaffected parents of ASD children (pASD) and 39 age-, sex-, and handedness-matched controls were enrolled. Multiple resting-state fMRI (rsfMRI) analyzing methods were applied, including amplitude of low-frequency fluctuation (ALFF), regional homogeneity (ReHo), degree centrality (DC), and functional connectivity (FC), to reveal the functional abnormalities of unaffected parents in ASD-related brain regions. Spearman Rho correlation analysis between imaging metric values and the severity of ASD traits were evaluated as well.

**Results:** ALFF, ReHo, and DC methods all revealed abnormal brain regions in the pASD group, such as the left medial orbitofrontal cortex (mOFC) and rectal gyrus (ROI-1), bilateral supplementary motor area (ROI-2), right caudate nucleus head and right amygdala/para-hippocampal gyrus (ROI-3). FC decreasing was observed between ROI-1 and right anterior cingulate cortex (ACC), ROI-2, and bilateral precuneus. FC enhancing was observed between ROI-3 and right anterior cerebellar lobe, left medial temporal gyrus, left superior temporal gyrus, left medial frontal gyrus, left precentral gyrus, right postcentral gyrus in pASD. In addition, ALFF values in ROI-1, DC values in ROI-3 were positively correlated with AQ scores in pASD (*ρ*_1_ = 0.298, *P*_1_ = 0.007; *ρ*_2_ = 0.220, *P*_2_ = 0.040), while FC values between ROI-1 and right ACC were negatively correlated with AQ scores (ρ_3_ = −0.334, *P*_3_ = 0.002).

**Conclusion:** rsfMRI metrics could be used as biomarkers to reveal the underlying neurobiological feature of ASD for unaffected parents.

## Introduction

Autism spectrum disorder (ASD) is a neurodevelopmental disorder characterized by social dysfunction, stereotyped behavior, and interest inhibition (Peng et al., 2020), which is more common in preschooler and may persist throughout life (Volkmar et al., 2005; Taylor et al., 2017). The pathogenesis of ASD is complex and considered to be the interaction between genes and environmental factors (Rossignol et al., 2014). Although the etiology is still unclear, twins and family studies have shown that ASD is highly heritable, with an estimated heritability as high as 90% (Lundström et al., 2012; Tick et al., 2016; Sandin et al., 2017). Relatives of ASD children who seem to be “normal” tend to behave with mild autistic traits, such as more “subthreshold” social skills, communication characteristics, and personalities than normal people (Dell’Osso et al., 2016). These subclinical features are defined as broader autism phenotypes (BAP; Folstein and Piven, 1991; Piven et al., 1997). Early studies have confirmed that autistic traits could be observed under the threshold in family members, especially in first-degree relatives (Hughes et al., 1997; Fiorentini et al., 2012), supporting the hypothesis that ASD has genetic predisposition (Page et al., 2016).

To date, there are no clear or definitive neuropathological findings in ASD. Autopsy and structural magnetic resonance imaging studies (sMRI) suggest that there is a pathological basis in the prefrontal cortex, amygdala-hippocampal complex, cerebellar cortex, basal ganglia nucleus, and anterior and posterior cingulate gyrus in ASD individuals (Amaral et al., 2008). Functional magnetic resonance imaging (fMRI) then revealed the functional alterations in these regions. For example, task-fMRI studies have shown that in tasks related to emotion regulation or social cognition, the brain areas that make up the social networks are less activated, including amygdala, temporoparietal junction, insular, and sub-frontal cortex (Pelphrey et al., 2007; Libero et al., 2014). The resting state functional magnetic resonance imaging (rs-fMRI) studies also detected the abnormal intrinsic functional connectivity between amygdala and suboccipital cortex/sensorimotor cortex in patients with ASD (Fishman et al., 2018), as well as the vital role of the default mode network (DMN) in the dimensions of communication and social interaction (Spencer et al., 2012; Xiao et al., 2021).

For biological parents of children with ASD, the investigators hypothesize that they may have an intrinsic pattern of impaired brain function similar to that of ASD individuals based on genetic hypotheses, and it has been confirmed by previous task- fMRI studies. Baron-Cohen et al. (2006) first evaluated the visual search and advanced emotion recognition task in first-degree relatives and found that compared to healthy controls, the unaffected parents showed decreased activities not only in the visual cortex during visual tasks but also in the middle temporal gyrus during emotion recognition tasks. As language disorder is another well-known feature of ASD. Wilson et al. (2013) explored the neuronal correlation of phonological processing skills in unaffected parents and controls. It suggested the differences in activated brain areas such as bilateral supplementary motor area (SMA), posterior cingulate cortex (PCC), thalamus, cerebellum, and midbrain. However, the task design usually needs to be based on the neuroanatomical hypothesis, that is, the altered brain function observed by task-fMRI may not be comprehensive due to the unclear and inconsistent neuropathological changes in ASD till recently. In addition, successful execution of tasks highly depends on parental cooperation, which may eventually affect the activation/deactivation results of the brain area. Therefore, it is necessary to adopt a more stable method to observe the brain function in unaffected parents.

Compared with task-fMRI, rs-fMRI has more advantages in reproducibility. However, the rs-fMRI characteristics still remain elusive in unaffected parents. Only a few family studies have reported rs-fMRI abnormalities in siblings of ASD children, for example, Lin et al. (2017) tested neuroanatomical endophenotypes and associated intrinsic functional connectivity (iFC) by comparing male youth with ASD, their unaffected brothers, and typically developed males (TD), and found that unaffected brothers experienced atypical gray matter volume reduction in the middle cingulate cortex as ASD patients compared with TD men, while iFC was reduced between middle cingulate cortex and right inferior frontal gyrus. Thus, rs-fMRI methods are valuable in revealing altered brain function in family members of ASD individuals. In this present study, we applied a variety of rs-fMRI data analyses, including the amplitude of low-frequency fluctuation (ALFF), regional homogeneity (ReHo), degree centrality (DC), and functional connectivity (FC) to observe whether unaffected parents have an anomalous rs-fMRI pattern similar to that of ASD individuals. Additionally, correlations between abnormal rs-fMRI results and autism-spectrum quotient (AQ; Baron-Cohen et al., 2001) score were explored to test whether the altered brain activities were associated with the severity of autistic traits.

## Methods and Materials

### Subjects

Forty-eight healthy parents of ASD children and 42 age-matched healthy parents of typically developed children were recruited. We used the Diagnostic and Statistical Manual of Mental Disorders (DSM-V; Battle, 2013) and Autism Behavior Checklist (ABC) to confirm the diagnosis of children with ASD. All diagnoses were made by experienced doctors in the department of child healthcare. After a detailed consultation, parents who encountered difficulties in daily life because of their intelligence level, or who were receiving treatment for any mental illness, were excluded from the study. None of the participants were clinically diagnosed as ASD. Data from nine adults were excluded due to head motion (either translation >1 mm or rotation >1° or both). Finally, we included 42 parents of children with ASD in the pASD group, and 39 parents of typically developed children as the control group (Table 1). All participants were recruited from Wuxi Children’s Hospital and Wuxi people’s Hospital. All the participants were right-handed and spoke Chinese as their first language. They filled out the AQ to measure the existence of autism traits. AQ is rated as a self-report questionnaire, with a total score ranging from 0 to 50. The higher a person’s AQ score, the closer their behavior is to an ASD individual. All participants provided a complete written informed consent to participate in the study, which was approved by the Ethics Committee of Wuxi people’s House Hospital. All methods are conducted in accordance with the guidelines and regulations of the Ethics Committee of Wuxi People’s Hospital.

**Table 1:** Demographic and clinical data of subjects.

**Characteristic**	**pASD (*n* = 42)**	**HC (*n* = 39)**	**Statistic**	***P* value**
Gender (male/female)	21/21	20/19	χ^2^ = 0.013	0.908
Age (years)	32.31 ± 6.90	33.03 ± 3.89	*t* = 0.569	0.571
AQ Score	16.26 ± 4.85	13.75 ± 3.50	*t* = 1.767	0.081
Head motion (mean FD)	0.08 ± 0.04	0.09 ± 0.03	*t* = 1.237	0.220

### Data acquisition

MRI data were collected in a SIEMENS 3T Trio scanner using a standard head coil. The participants were instructed to rest with their eyes closed and keep still. A foam pad was used to minimize the head motion. The rs-fMRI data were acquired using gradient echo type echo planar imaging (GRE-EPI) sequence with the following parameters: TR/TE = 2,000 ms/30 ms; FA = 90°; matrix = 64 × 64; FOV = 240 × 240 mm^2^; slice thickness = 4 mm; and slice gap = 0.4 mm. A total of 33 axial slices were used to encompass the whole brain. Each section contained 250 volumes (500 s in total). Conventional MRI brain images such as T1WI, T2WI, DWI, and T2-FLAIR were collected to exclude anatomic abnormalities, and parameters were listed as follows, T1WI: TR/TE = 500 ms/8.5 ms; matrix = 120 × 256; FOV = 230 × 230 mm^2^; slice thickness = 5 mm; slice gap = 1.5 mm; slice number = 20. T2WI: TR/TE = 5,000 ms/117 ms; matrix = 248 × 320; FOV = 220 × 220 mm^2^; slice thickness = 5 mm; slice gap = 1 mm; slice number = 20. DWI: TR/TE = 5,100 ms/100 ms; matrix = 192 × 192; FOV = 230 × 230 mm^2^; slice thickness = 5 mm; slice gap = 0.4 mm; slice number = 20; b-value = (1,000). T2-FLAIR: TR/TE = 8,000 ms/94 ms; matrix = 186 × 256; FOV = 230 × 230 mm^2^; slice thickness = 5 mm; slice gap = 1 mm; slice number = 20.

## Data Preprocessing

The rs-fMRI data were preprocessed using the toolkit of Data Processing Assistant for Brain imaging (DPABI_V6.1_220101; Yan et al., 2016), a MATLAB (R2018b) Toolbox for “Pipeline” data analysis of functional images. The first 10 volumes of each participant were removed. The remaining 240 scans were slice-time corrected and then realigned to the first volume to correct the head motions. No translation or rotation parameters in any given dataset exceeded ±1 mm or ±1°. All realigned images were warped into a standard stereotaxic space at a 3 × 3 × 3 mm^3^ resolution, according to the Montreal Neurological Institute (MNI) echo-planar imaging template. A detrending was performed after regressing out the nuisance covariates, including white matter, cerebrospinal fluid, and global signals.

### ALFF calculation

The data were spatially smoothed by convolution with a 6 mm FWHM (full width at half maximum) isotropic Gaussian kernel, to reduce the influence of deformation and noise in the standardization process, improve the signal-to-noise ratio and statistical efficiency, and enhance the image effect. Then band-pass filtering (0.01–0.08 Hz) is applied to the time series to eliminate the influence of low-frequency drift and high-frequency noise. ALFF is calculated based on the fast Fourier transform (FFT), and the time series of each voxel is transformed into the frequency domain without bandpass filtering. The square root of each frequency of the power spectrum is calculated, and then the mean square root of each voxel in the 0.01–0.08 Hz band is obtained. The last step is to divide the ALFF of each voxel by the global average of ALFF values (mALFF; Zang et al., 2007).

### ReHo calculation

The ReHo brain map was constructed by calculating the Kendall coefficient of time series consistency between each voxel and its neighboring voxels. The average ReHo value of the whole brain was subtracted from that of each voxel and then divided by the standard deviation for subsequent analysis. The KCC-ReHo values of all individual voxel directions are calculated and normalized to the KCC-ReHo Z-value (Zuo et al., 2013). Finally, a 6 mm FWHM Gaussian kernel is used for smoothing to make a comparison between groups.

### DC calculation

For each voxel, the time process of the time series is extracted, and the Pearson correlation coefficient with other voxels in the brain is calculated. The Pearson correlation coefficient matrix between any pair of voxels was obtained to construct the whole brain functional connection matrix of each participant. Finally, the binary DC was calculated and then smoothed with a 6 mm FWHM Gaussian kernel for inter-group comparison (Zuo et al., 2012).

### Inter-group analysis and FC calculation

The above three rs-fMRI metrics (ALFF, ReHo, DC) were evaluated by a two-sample *t*-test, and the cluster that survived was selected as seed to calculate FC based on a whole brain voxel level. Subsequently, the FC value after Z transformation was evaluated by a two-sample *t*-test to observe whether there is a surviving cluster. The statistical results were regressed by sex, age, and head motion (using mean FD), and corrected using GRF (voxel *P* < 0.005, cluster *P* < 0.05).

### Statistical analysis between imaging and clinical information

ALFF, ReHo, DC, and FC values were extracted from the corresponding survival cluster. Spearman Rho correlation analysis was performed between the above metric value and AQ score, respectively. All the results were considered significant at *P* < 0.05.

## Results

### Analysis results of rs-fMRI metrics

**Figure 1:**
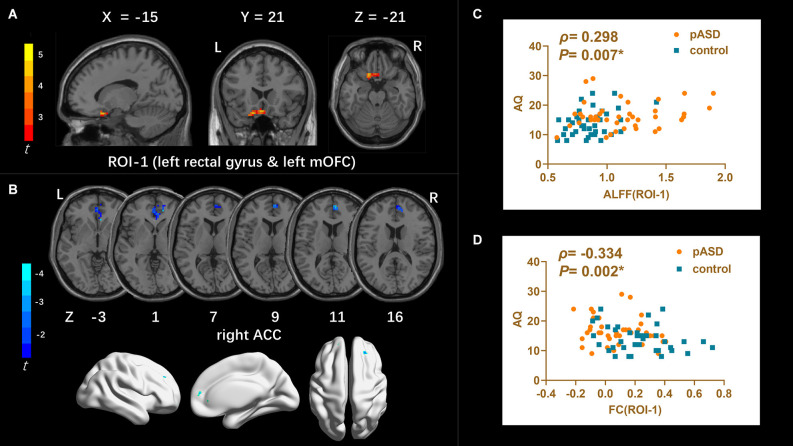
**(A)** Intergroup comparison of ALFF: Compared with the control group, ALFF was significantly increased in the left mOFC and rectal gyrus (ROI-1) in pASD than that of the control group [Peak MNI coordinate = (−3, 21, −18), Peak intensity = 3.77]. **(B)** Intergroup comparison of FC based on ALFF seed (ROI-1): FC in right ACC was significantly attenuated in pASD compared with the controls [Peak MNI coordinate = (6, 51, 12), Peak intensity = −3.86]. The results of **(A,B)** were regressed for sex, age, and head motion, and were corrected using GRF (voxel *P* < 0.005, cluster *P* < 0.05). The second row of pictures in **(B)** presented the projection of the FC results onto the cortical surface. **(C,D)** Spearman Rho correlation analysis: the mALFF values of ROI-1 were positively correlated with AQ scores (*ρ* = 0.298, *P* = 0.007); zFC values between ROI-1/right ACC were negatively correlated with AQ scores (*ρ* = −0.334, *P* = 0.002). The results of **(C,D)** were considered statistically different at *P* < 0.05. Abbreviation: mOFC, medial orbitofrontal cortex; ACC, anterior cingulate cortex; mALFF, mean ALFF; zFC, z-transformed FC.

**ALFF:** Compared to the control group, ALFF increased significantly in the left medial orbitofrontal cortex (mOFC) and left rectal gyrus in pASD (Table 2). The cluster was marked as ROI-1 (Figure 1A).

**Table 2:** Brain regions with significant differences of ALFF, ReHo, DC, and FC between the pASD and control group.

**Metrics**	**GRF correction**				**Cluster Size (voxles)**	**Peak MNI coordinates**			**Brain region (AAL)**	**Peak intensity (*T*-score)**
	**FWHM (x, mm)**	**FWHM (y, mm)**	**FWHM (z, mm)**	**Minimum cluster sizev**		**X**	**Y**	**Z**
pASD>control
ALFF	5.49	3.63	3.72	10	74	−3	21	−18	medial orbitofrontal cortex and rectal gyrus_L	3.77
ReHo	8.35	9.04	8.59	60	175	−6	6	60	supplementary motor area_L	4.27
DC	8.87	9.38	8.72	66	108	12	18	3	caudate head_R, extend to amygdala_R &parahippocampa gyrus_R	4.17
FC (ROI-3)	11.24	12.83	11.05	121	Cluster 1 414	12	−51	−15	cerebellum_R	4.78
					Cluster 2 382	−54	−45	0	superior temporal gyrus_L	4.37
					Cluster 3 334	−9	−21	51	supplementary motor area_L	4.27
					Cluster 4 579	−39	−15	51	precentral gyrus_L	4.45
					Cluster 5 297	39	−33	66	postcentral gyrus_R	4.67
pASD<control
FC (ROI-1)	8.29	8.68	7.84	54	59	6	51	12	anterior cingulate cortex_R	−3.86
FC (ROI-2)	8.36	8.78	7.82	54	110	0	−72	48	bilateral precuneus	−3.48

ROI-1, left medial orbitofrontal cortex and rectal gyrus; ROI-2, bilateral supplementary motor areas (SMA); ROI-3, right caudate head, right amygdala, and para-hippocampal gyrus.

**Figure 2:**
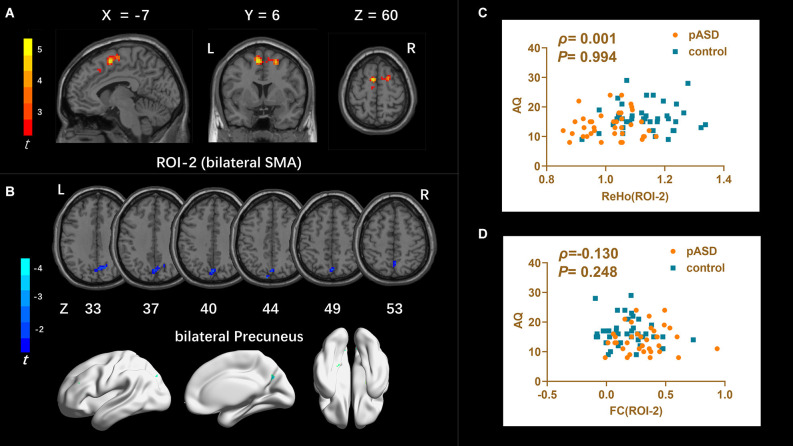
**(A)** Intergroup comparison of ReHo: compared with the control group, the ReHo values of bilateral supplementary motor areas (SMA) (ROI-2) of pASD were significantly higher than those of the control group [Peak MNI coordinate = (−6, 6, 60), Peak intensity = 4.27]. **(B)** Intergroup comparison of FC based on ReHo seed (ROI-2): compared with the controls, FC in the bilateral precuneus of pASD was significantly decreased [Peak MNI coordinate = (0, −72, 48), Peak intensity = −3.48]. The results of **(A,B)** were regressed for sex, age, and head motion, and were corrected using GRF (voxel *P* < 0.005, cluster *P* < 0.05). The second row of pictures in **(B)** presented the projection of the FC results onto the cortical surface. **(C,D)** Spearman Rho correlation analysis: the result between smoothed mReHo values of ROI-3 and AQ scores was *ρ* = 0.001, *P* = 0.994, and the result between zFC value of ROI-3/right ACC and AQ score was *ρ* = 0.130, *P* = 0.248. Neither result of **(C)** or **(D)** was considered statistically different at *P* < 0.05. Abbreviation: SMA, supplementary motor areas; mReHo, mean ReHo; zFC, z-transformed FC.

**ReHo**: The ReHo values of SMA on both sides of pASD were significantly higher than those in the control group (Table 2). The cluster was marked as ROI-2 (Figure 2A).

**Figure 3:**
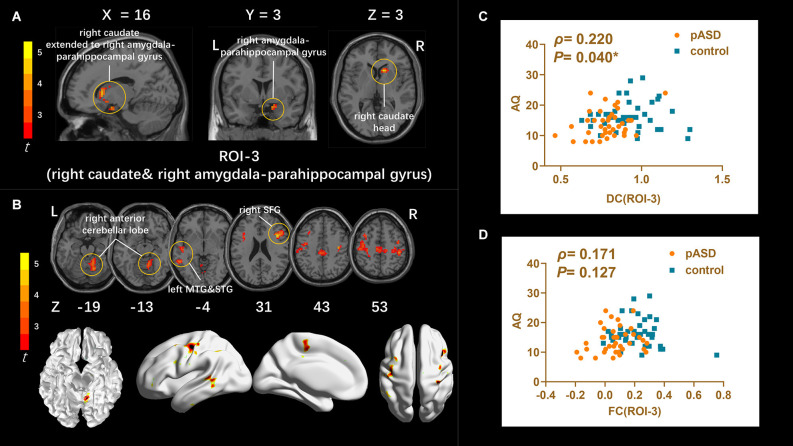
**(A)** Intergroup comparison of DC: DC values were significantly higher in the right caudate head, right amygdala, and para-hippocampal gyrus (ROI-3) of pASD compared with controls [Peak MNI coordinate = (12, 18, 3), Peak intensity = 4.17]. **(B)** Intergroup comparison of FC based on DC seed (ROI-3): compared with normal controls, pASD showed significantly enhanced FC in right anterior cerebellar lobe, left MTG, left STG, left MFG, left precentral gyrus, and right postcentral gyrus [Peak MNI coordinate = (12, −51, −15), Peak intensity = 4.78]. The results of **(A,B)** were regressed for sex, age, and head motion, and corrected using GRF (voxel *P* < 0.005, cluster *P* < 0.05). The second row of pictures in **(B)** presented the projection of the FC results onto the cortical surface. **(C,D)** Spearman Rho correlation analysis: the smoothed mDC values of ROI-3 were positively correlated with AQ scores (*ρ* = 0.220, *P* = 0.040). While the results between zFC values (generated from ROI-3) and AQ scores was *ρ* = 0.171, *P* = 0.127, which was not statistically correlated. The results of **(C,D)** were considered statistically different at *P* < 0.05. Abbreviation: MTG, medial temporal gyrus; STG, superior temporal gyrus; MFG, medial frontal gyrus; mDC, mean DC; zFC, z-transformed FC.

**DC:** The DC values of the right caudate nucleus head, right amygdale, and para-hippocampal in pASD were significantly higher than those in the control group (Table 2). The cluster was marked as ROI-3 (Figure 3A).

**FC**: For ROI-1 (left mOFC and rectal gyrus), the FC of pASD in the right anterior cingulate cortex (ACC) was significantly lower than that in the control group (Figure 1B, Table 2). For ROI-2 (bilateral SMA), the FC of pASD in the bilateral precuneus was significantly decreased compared with the control group (Figure 2B, Table 2). For ROI-3 (right caudate nucleus head, right amygdale, and para-hippocampal gyrus), the FC of pASD was significantly enhanced in the right anterior cerebellar lobe, left medial temporal gyrus (MTG), left superior temporal gyrus (STG), left medial frontal gyrus (MFG), left precentral gyrus, and right postcentral gyrus, compared with normal controls (Figure 3B, Table 2).

### Analysis results between imaging and clinical information

The mALFF value of ROI-1 (left mOFC and rectal gyrus) was positively correlated with AQ score (*ρ* = 0.298, *P* = 0.007; Figure 1C). The DC value of ROI-3 (right caudate nucleus head, right amygdala, and para-hippocampal gyrus) was positively correlated with the AQ score (*ρ* = 0.220, *P* = 0.040; Figure 3C). The FC value between ROI-1 (left mOFC and rectal gyrus) and right ACC was negatively correlated with AQ score (*ρ* = −0.334, *P* = 0.002; Figure 1D). Meanwhile, there was no correlation between ReHo value of ROI-2 (bilateral SMA) and AQ score (Figure 2C). FC results calculated from either ROI-2 or ROI-3 were not correlated with AQ scores (Figures 2D, 3D).

## Discussion

One of our aims was to investigate whether resting brain function differs between unaffected parents with ASD children and parents with typically developing children. By calculating rs-fMRI metrics including ALFF, ReHo, DC, we found that spontaneous activity in left mOFC and rectal gyrus, bilateral SMA, right caudate nucleus head, right amygdala, and para-hippocampal gyrus was significantly enhanced in unaffected parents compared with normal controls. Abnormalities in whole-brain voxel level were also present in FC results based on the brain regions described above. In addition, we found that brain regions with abnormally increased ALFF and DC values were positively correlated with AQ scores; while FC values based on ALFF results were negatively correlated with AQ scores. Correlation analysis results indicated that unaffected parents of children with ASD may have subclinical elevations in ASD traits.

One of the core symptoms in ASD individuals is social deficits, and the amygdala plays an important role in social and emotional interactions as a key component of the social brain (Hadjikhani et al., 2017; Lassalle et al., 2017). ASD-related abnormalities in the amygdala have already been reported in studies focusing on assessing functions such as facial and emotional expression (Baron-Cohen et al., 2000). However, other studies have also found the deactivation of the amygdala in ASD (Pierce and Redcay, 2008; Lassalle et al., 2017). Therefore, mechanistic abnormalities associated with the amygdala remain to be elucidated. A meta-analysis of ts-fMRI (Peng et al., 2020) indicated abnormal activation in social brain regions in ASD individuals compared with controls, featured by a characteristic over-activation of the right amygdala. This is consistent with the findings of the DC approach in this study. DC is a data-driven method based on Eigenvector Centrality Mapping (ECM), which can accurately and objectively detect all brain regions as communication hubs, and these hubs have stronger connectivity with other parts of the brain (Lohmann et al., 2010). We observed higher DC in the right amygdala and para-hippocampal gyrus in unaffected parents than in controls, suggesting that this node may also be an important communication hub in pASD. In addition, the global FC results based on right amygdala and para-hippocampal gyrus in this study further showed wide range of long-distance FC abnormalities by this central hub, such as enhanced FC with anterior cerebellar lobe, left MTG, left STG, left MFG, left precentral gyrus, and right postcentral gyrus. Functional connectivity abnormalities involved in these brain regions might be the basis of visuomotor deficits in ASD individuals (Guo et al., 2017). Therefore, we speculate that similar to ASD individuals, the centrality of the right amygdala and para-hippocampal complex and its FC pathways may be responsible for causes if the parents present subthreshold visual deficits related to ASD. Furthermore, as ASD individuals typically demonstrate enhanced short-distance FC and diminished long-distance FC when performing tasks (May and Kana, 2020), enhanced long-distance FC is more likely to reflect functional compensation of unaffected parents on this pathway. We also found the increased DC value in the right caudate head of pASD, indicating the importance of this hub. Anatomically, the caudate head is adjacent to the amygdala, so there might be a structural basis that partially coincides with the functional connectivity pathway in which the amygdala resides (Jung et al., 2013). Caudate head participates in the regulation of the reward circuit (Dichter et al., 2012; Kohls et al., 2013), and meta-analyses on social reward mechanisms have shown that ASD individuals experience a large number of missing clusters, as shown by hypoactivation, in social reward circuits in the bilateral caudate head during social stimulation test and are negatively correlated with the social scale rating in ASD individuals (Clements et al., 2018). Although only the abnormality of right caudate head was observed in this study, the positive correlation between DC value and AQ score suggested that dysregulation in the reward circuit of social stimuli may have emerged in pASD compared with the controls.

The medial prefrontal cortex (mPFC) is also crucial in the social brain, and mOFC is one of the most repeatedly studied mPFC structures (Mori et al., 2013; Jonker et al., 2015). mOFC plays a significant role in the process of social reinforcement and reward processes, and its impairment not only affects the completion of executive task function but also affects cognitive integrity (Pina-Camacho et al., 2012; Nestor et al., 2013). Executive disability is one of the major manifestations in ASD individuals (Happé and Frith, 2006), and meta-analysis studies have revealed that ASD individuals have significantly enhanced activation of the mOFC when performing various tasks associated with executive function, such as attention pointing and shifting, spatial attention tasks, and key press responses, compared with normal controls (Schmitz et al., 2006). In this present study, we found that ALFF was increased in the left mOFC of pASD compared with controls at rest, which implies the spontaneous activity of local neurons is significantly enhanced, suggesting that its executive function may have been affected and necessitating stronger spontaneous brain activity to cope. Further rsFC results based on left mOFC showed that pASD had significantly attenuated rsFC in right ACC, however, none of the available studies reported the mOFC/ACC pathway separately in ASD individuals. The decreased rsFC between left mOFC/right ACC found in this study reflects abnormal functional connectivity between the left and right cerebral hemispheres. Based on the cortical underconnectivity theory, aberrant patterns of rsFC in ASD can be described as local overconnectivity, whereas long-distance as underconnectivity (Courchesne and Pierce, 2005; Lau et al., 2019). As a typical long-distance connection, interhemispheric rsFC can be used as an important indicator to describe interhemispheric synchronization abnormalities in spontaneous neural activity in ASD. Abnormal rsFC across hemispheres in pASD presented in this study suggests that the medial prefrontal cortex, particularly the left mOFC, of unaffected parents, has decreased synchronization of long-distance spontaneous neuronal activity with the right ACC, which is similar to interhemispheric rsFC patterns in typical ASD individuals (Lau et al., 2019). In addition, early developmental impairment of the orbitofrontal-amygdala loop is critical to brain injury in ASD individuals, and both socio-emotional cognitive and self-regulation behavioral disorders are associated with deficits in this circuit (Watanabe et al., 2014). However, no abnormal rsFC between mOFC and amygdala was observed in pASD in this study, which suggests that the social cognition and behavior norms of unaffected parents may still be regarded as normal.

A quantitative meta-analysis of rs-fMRI in ASD individuals showed that the spontaneous activity of right SMA, middle frontal gyrus, inferior frontal gyrus, left precentral gyrus, and bilateral cerebellar hemispheric lobules (VIII/IX) in ASD patients was significantly higher than that of the controls (Wang et al., 2018). In this study, by applying the ReHo method, it was also found that the local consistency of bilateral SMA in pASD was significantly higher than that in the controls, indicating that the abnormal spontaneous activity of SMA may be a common rs-fMRI feature of ASD individuals and unaffected parents (Maximo et al., 2013; Itahashi et al., 2015; Dajani and Uddin, 2016). SMA is responsible for planning and organizing movements, especially visual movements that require selection and execution (Mosconi and Sweeney, 2015). ASD individuals experience general motor control and motor coordination dysfunction in addition to core symptoms such as social impairment (Gallese et al., 2013), therefore, functional abnormalities in SMA are likely to be associated with the aforementioned motor disorders. However, the neural mechanisms involved in motor dysfunction in ASD are unclear (Mosconi and Sweeney, 2015). A task-fMRI study based on the visual motor task paradigm (Lepping et al., 2022) showed that ASD individuals had an equal proportion of significantly increased activation in bilateral SMA, bilateral superior parietal lobule, and contralateral middle/inferior frontal gyrus with increasing visual gain, indicating that the ability to process increased visual feedback was compromised. Meanwhile, FC between parietal sensory processing and frontal cortex motor control area was weakened during behavior, suggesting that the functional integration of the parietal lobe and frontal lobe region was lower in ASD individuals. The decreased rs-FC in SMA/precuneus in pASD derived from this study has some similar characteristics to the above results, that is, the degree of functional integration in parietal and frontal lobe was reduced, and it is speculated that unaffected parents may be more likely to have intrinsic functional abnormalities in motor selection and execution. At the same time, rs-FC between SMA and precuneus belongs to long-distance connectivity, and the underconnectivity of this pathway shown by pASD has a similar impaired pattern as the long-distance under connectivity of rsFC reported for ASD individuals (Courchesne and Pierce, 2005; Lau et al., 2019).

In the present study, ALFF values in the left mOFC and rectal gyrus, DC values in the right caudate head and the right amygdala/para-hippocampal gyrus were associated with higher AQ scores in pASD, which referred to higher level of autistic traits. Both ROIs belong to prominent hubs of the social brain network (Sato and Uono, 2019), and it was speculated that pASD was likely to have subthreshold social disorders. On the other hand, there was a negative correlation between rs-FC results based on left mOFC/rectal gyrus and AQ scores, suggesting that this brain region may be more likely to reflect the underlying neurobiological features of ASD.

Limitations: In the current study, we focused on the changes in rs-fMRI patterns of unaffected parents with ASD children, and in the future, we need to investigate the potential coupling alterations in structure-function in key brain regions of unaffected parents. In addition, further studies are needed to investigate the relationship between neuroimaging and gene expression to provide more conclusive evidence for searching subthreshold ASD-related neuroimaging endophenotype.

## Conclusion

We identified regional spontaneous brain activity abnormalities in multiple brain regions in unaffected parents with ASD children, as well as corresponding rsFC changes, by analyzing multiple rs-fMRI metrics. These abnormal brain regions all belong to the important nodes of brain damage in ASD individuals, and the abnormal patterns of rsFC analyzed by these brain regions also have similarities with the impaired patterns in ASD individuals. Particularly, spontaneous brain activity in the left mOFC and the left rectal gyrus, as well as the global FC generated from this node, were associated with higher level of autistic traits, indicating that rs-fMRI abnormalities in this area may be more reflective of a underlying neurobiological feature of ASD for unaffected parents.

## Data Availability Statement

The original contributions presented in the study are included in the article, further inquiries can be directed to the corresponding author/s.

## Ethics Statement

The studies involving human participants were reviewed and approved by Wuxi People’s Hospital Clinical New Technology and Scientific Research Ethics Committee. The patients/participants provided their written informed consent to participate in this study.

## Author Contributions

X-WZ, L-LZ, Z-MZ, L-YW, Z-XD, and X-MF contributed to data collection, data and statistical analyses, and wrote the manuscript. All authors contributed to the article and approved the submitted version.

## Funding

This work was supported by Department of Health of Zhejiang Province (grant numbers: 2021418656 and 2022519634).
